# Fabrication of Biocompatible Helical Fibers Using an Optical Vortex Beam

**DOI:** 10.1002/asia.202500361

**Published:** 2025-06-10

**Authors:** Kenta Homma, Yoshihisa Matsumoto, Yasushi Tanimoto, Kyoko Masui, Chie Hosokawa, Takashige Omatsu, Michiya Matsusaki

**Affiliations:** ^1^ Division of Applied Chemistry Graduate School of Engineering Osaka University 2‐1 Yamadaoka Suita Osaka 565–0871 Japan; ^2^ Frontier Research Base for Global Young Researchers Graduate School of Engineering Osaka University 2‐1 Yamadaoka Suita Osaka 565–0871 Japan; ^3^ Department of Chemistry Graduate School of Science Osaka Metropolitan University 3‐3‐138 Sugimoto Sumiyoshi‐ku Osaka 558–8585 Japan; ^4^ Graduate School of Engineering Chiba University 1–33 Yayoi‐cho Inage‐ku Chiba 263–8522 Japan; ^5^ Molecular Chirality Research Center Chiba University 1–33 Yayoi‐cho Inage‐ku Chiba 263–8522 Japan

**Keywords:** Helicity, Optical angular momentum, Optical vortex, Photopolymerization, Poly(ethylene glycol)

## Abstract

Helical structures are a fundamental characteristic of biological tissues, yet helical biomaterial scaffolds remain underdeveloped. Optical vortex beams, a unique class of light with helical wavefronts, carry optical angular momentum (OAM). Interestingly, it has been discovered that the OAM of optical vortex beams twists the irradiated photocurable resins to form helical fiber structures. This phenomenon opens up new possibilities that optical vortex beams enable the creation of photopolymerized structures for tissue engineering scaffolds. However, the fabrication of helical fibers formed of biocompatible polymers has not been established yet. In this study, we successfully fabricated helical gel fibers using poly(ethylene glycol) (PEG), a representative biocompatible polymer, through photopolymerization with an optical vortex beam. The helical wavefront of the optical vortex beam enabled the creation of twisted PEG gel microscale fibers with minimal branching, likely due to the OAM transferred to the gel precursors during photopolymerization. In contrast, PEG gel microscale fibers fabricated using a Gaussian beam with a planar wavefront exhibited significant branching. These findings demonstrate the potential of optical vortex beams for fabricating helical structures with biocompatible polymers, offering a promising approach for applications such as helical tissue engineering.

## Graphical abstract

A helical gel fiber was fabricated through the photopolymerization of poly(ethylene glycol) using an optical vortex beam. The helical structure was imparted during photopolymerization because of the orbital angular momentum of the optical vortex beam. The helical gel fiber contributes to the elucidation of the structure‐function relationship in cellular tissues.

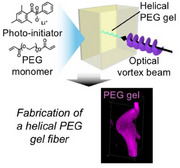

## 1 Introduction

The ultimate goal of tissue engineering is to construct tissues and organs with sophisticated structures and functions comparable to those of the human body.^[^ ^1^, ^2^ ^]^ This field has significantly advanced our understanding of how biophysical and biochemical factors in the cellular microenvironment, or the extracellular matrix, influence cellular behavior.^[^ ^3^ ^]^ However, creating *in vitro* organs or tissues with both complex structures and functions remains a challenge. While efforts have been made to elucidate the underlying mechanisms governing the fabrication of functional tissues,^[^ ^3^ ^]^ the effects of helical geometry have rarely been explored in tissue engineering. Helical structures are a fundamental feature of biological tissues, as exemplified by the spatial arrangement of vascular smooth cells^[^ ^4^ ^]^ and the helical ventricular myocardial band of the heart.^[^ ^5^ ^]^ The development of helical biomaterial scaffolds could provide significant insights into how such structures influence cellular and tissue‐level functions.

Hydrogels are ideal candidates for tissue engineering scaffolds. Conventional methods for fabricating hydrogels with specific structures rely on molds, where pre‐gel solutions are filled and polymerized or crosslinked through spontaneous or thermally initiated reactions. However, mold‐based methods often face challenges, such as the difficulty of removing intact hydrogels due to adhesion and fragility. In contrast, photolithography enables the fabrication of hydrogels with intricate 3D structures without molds. Advanced photolithographic techniques, including digital light processing^[^ ^6^ ^]^ and multiphoton lithography based on photoinitiated chemistry,^[^ ^7^ ^]^ have demonstrated the ability to create precise hydrogel structures for tissue engineering scaffolds. However, these layer‐by‐layer additive manufacturing techniques are time‐consuming with low throughput.

**Figure 1:**
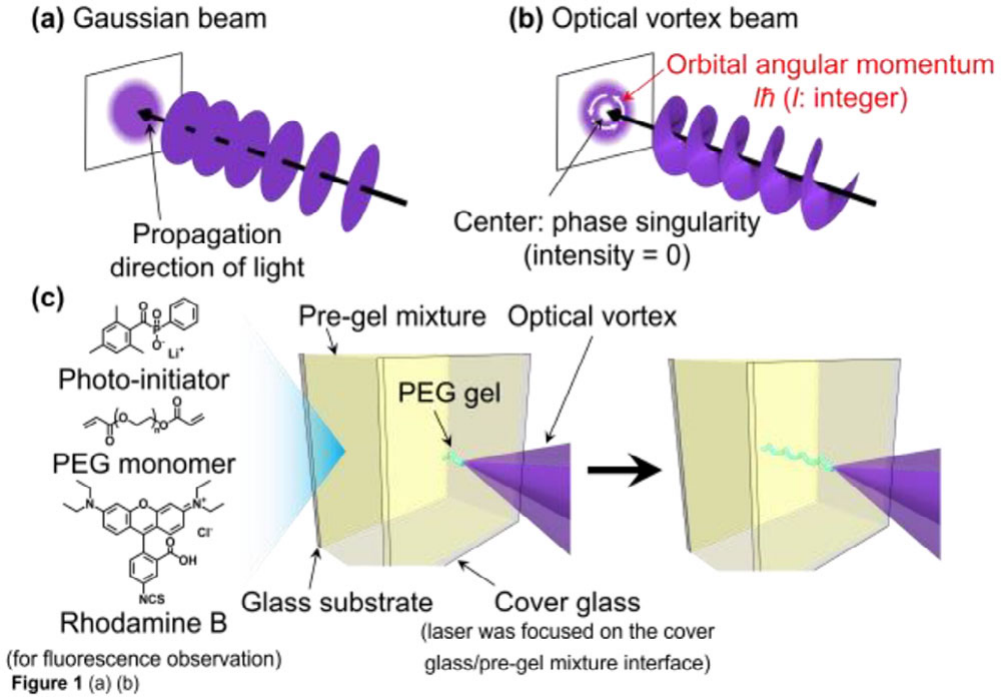
(a,b) Schematic illustrations of the propagating optical waves of (a) the Gaussian beam and (b) the optical vortex beam. (c) Conceptual illustration of the helical gel fiber fabrication by the photopolymerization using an optical vortex beam. An optical vortex beam is focused onto the interface between the pre‐gel mixture and a cover glass.

Unlike a conventional Gaussian beam with a planar wavefront, an optical vortex beam possesses a unique annular spatial form and azimuthal radiation force, referred to as the orbital angular momentum (OAM), determined by the topological charge *l*, which indicates the winding number (an integer) of its helical wavefront^[^ ^8^ ^]^ (Figure 1a,b). In addition, the handedness of the optical vortex beam is determined by the positive or negative sign of integer *l*. Recently, it has been demonstrated that the OAM of an optical vortex beam can “twist” a photocurable resin (NOA63, Norland Optics Adhesive 63) to produce millimeter‐scale helical fibers through photopolymerization in less than 1 s and at high throughput.^[^ ^9^ ^]^ In addition, higher‐order optical vortex beams with a topological charge *l* > 1 enable the fabrication of helical fibers with multiple branches. The diameter and helical pitch of these fibers typically range from 1–5 microns and several hundred microns, respectively, matching the dimensions of cells and tissues. This suggests that optical‐vortex‐induced photopolymerization holds promise for creating helical fibers from biocompatible materials for tissue engineering scaffolds. In this study, we present the first demonstration of helical poly(ethylene glycol) (PEG) gel fibers fabricated using an optical vortex beam (Figure 1c). Optical vortex beams with topological charges *l* of +1 and ±4 were irradiated onto pre‐gel solutions containing PEG (a biocompatible polymer), a photoinitiator, and rhodamine B to produce fluorescent PEG fibers. The 3D structure of the PEG fibers was captured from the reddish emission of the encapsulated rhodamine B using a laser‐scanning confocal microscope.

## 2 Results and Discussion

**Figure 2:**
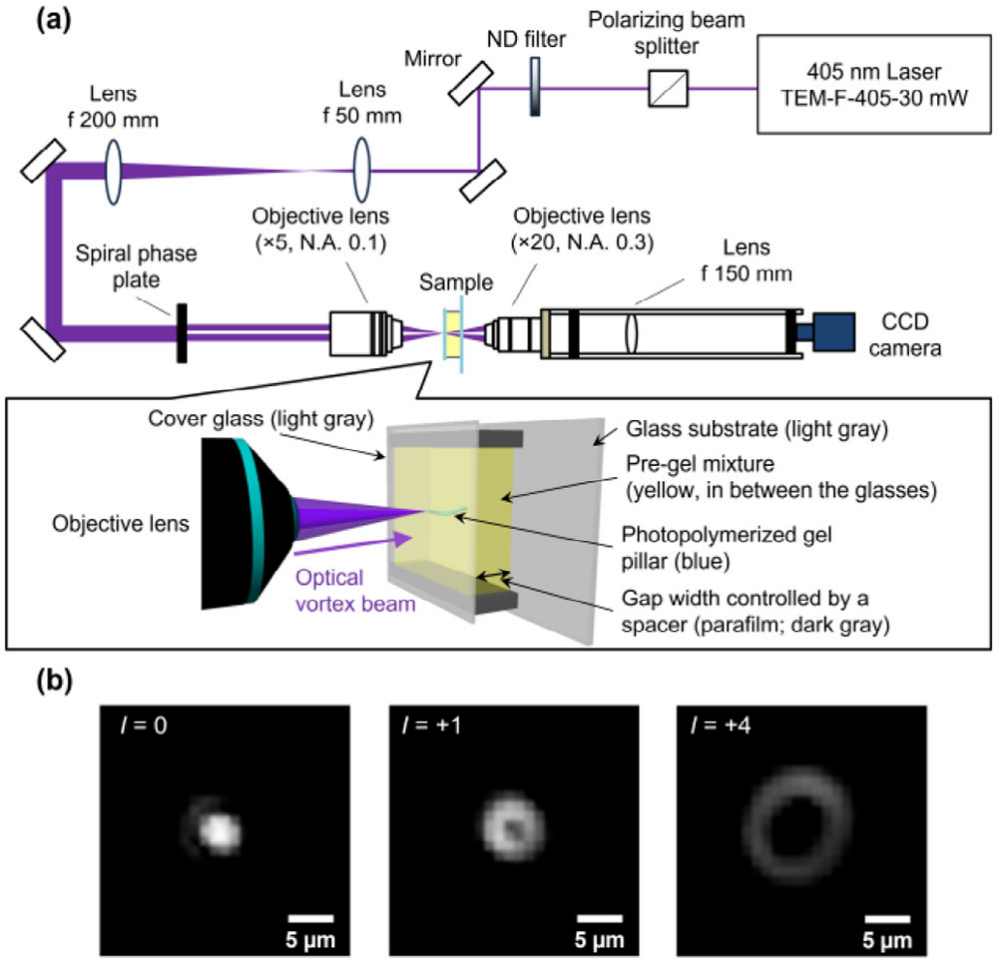
(a) Schematic of the optical setup. Using the ×5 objective lens, the laser beam was focused on the interface between the pre‐gel mixture and the cover glass closer to the × 5 objective lens. (b) Transmitted images captured at the focus of the lasers. *l* in the top‐left corner of each image represents the topological charge of the irradiated beam.

Figure 2a illustrates the experimental setup for photopolymerization. The sample consisted of a pre‐gel mixture water suspension containing 50 mM lithium phenyl‐2,4,6‐trimethylbenzoylphosphinate (LAP) as the photoinitiator, 10 wt% poly(ethylene glycol)diacrylate (PEGDA),^[^ ^10^, ^11^ ^]^ and 0.16 mM rhodamine B isothiocyanate (for fluorescence observation after fabrication). LAP allows photopolymerization even in the presence of cells while maintaining high cell viability.^[^ ^12^ ^]^ A 405 nm continuous‐wave laser was used to polymerize PEGDA using LAP as an initiator (Supporting Information, Figure S1a,b), and its output was converted into an optical vortex with topological charges *l* of 0 (Gaussian beam), +1, and ± 4 by employing a spiral phase plate. The generated optical vortex was focused by an ×5 objective lens to be an annular spot with diameters of 3.6 µm (*l* = 0), 6.3 µm (*l* = +1), and 11.3 µm (*l* = +4). The exposure time was fixed at 10 s. Notably, the experimentally focused spot diameters aligned well with the theoretical scaling formula $\sqrt{|l|+1}$ (Table 1).^[^ ^13^ ^]^ As shown in Figure 2b, the transmitted optical vortex beam typically exhibits an annular spatial form with an on‐axis phase singularity in the absence of a sample. The incident laser power on the sample was controlled to 0.8–0.9 µW (Video S1, Supporting Information). Upon laser irradiation on the sample (thickness: 300–400 µm), photopolymerization‐induced gelation occurred immediately, forming a PEGDA gel at the focal point of the interface between the cover glass and the pre‐gel mixture. Interestingly, the phase singularity of the transmitted beam began rotating clockwise (Video S1) with a short time lag (within 1 s), indicating that the PEGDA gel twisted during photopolymerization due to OAM transfer effects.^[^ ^9^, ^14^ ^]^ This rotation of the phase singularities gradually dampened, resulting in the formation of a helical PEGDA gel fiber structure, as previously reported by Lee et al.^[^ ^9^, ^14^ ^]^

**Table 1:** Characterizations of the laser beams with different topological charges.

	Topological Charge *l*		
	0	+1	+4
Laser power (µW)	0.86	0.89	0.82
Experimental beam diameter at focal spot (µm)	3.6	6.3	11.3
Theoretical beam diameter at focal spot (µm)	4.9	7.0	11.0
Confocal length (µm)	86	172	430

Bulk photopolymerization of the pre‐gel solution was also attempted under equivalent light intensity (1.0 × 10^3^ mW cm^−2^) and irradiation duration (10 s). From the infrared transmittance measurements (Figure S1c, Supporting Information), peaks derived from C─H (2875 cm^−1^), C═O (1729 cm^−1^), and C─O (1095 cm^−1^) bonds were confirmed from PEGDA monomer and photopolymerized PEGDA bulk hydrogel. There was no peak for C═C stretching (1633 cm^−1^) for the PEGDA gel, which suggested the disappearance of acrylate C═C bonds and that the polymerization took place. However, C═C stretching was also barely recognizable for the PEGDA monomer. This may be due to the weak infrared activity of the C═C bonds and the large molecular weight of the PEGDA monomer (6,000 g mol^−1^). For the rheological measurements (Figure S1d, Supporting Information), the storge modulus *G*’ and the loss modulus *G*’’ significantly increased after the photoirradiation due to the crosslinking between the PEGDA monomers. Furthermore, *G’* exceeded *G’’* which indicated the formation of the PEGDA gel.

**Figure 3:**
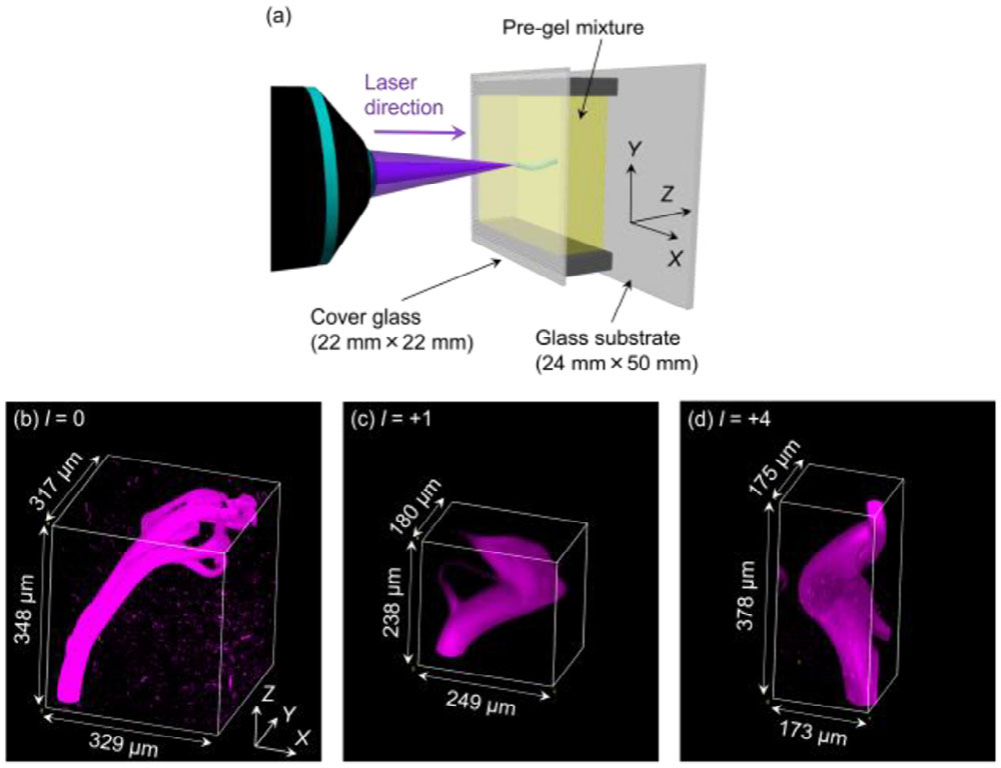
(a) Illustration of the photopolymerization by the optical vortex beam and the xyz directions corresponding to the xyz axis in (b–d). (b–d) Confocal fluorescence images of the Rhodamine B encapsulated inside the PEGDA gels. The values on the upper left of each image in black rectangles represent the topological charge *l* of the optical vortex beam used to fabricate each PEGDA gel (*l* = 0, +1, and +4).

The PEGDA gels photopolymerized in Figure 2 were observed using a confocal microscope. As illustrated in Figure 3a, the 22 mm × 22 mm cover glass (where the laser beam was focused during photopolymerization) was placed in contact with the microscope stage. Figure 3b–d present confocal fluorescence images of rhodamine B (fluorescence spectra shown in Figure S1e) encapsulated within the photopolymerized PEGDA gels. In all cases, the photopolymerized PEGDA gels bridged the two glass surfaces. Each PEGDA gel was rotated to better understand its 3D structure (Video S2). When irradiated with a Gaussian beam (*l* = 0, Figure 3b), the PEGDA gel did not stand perpendicular to the cover glass. This was likely due to the sample being set perpendicular to the optical table while the laser beam was irradiated horizontally during photopolymerization (Figure 3a). As the PEGDA gel formed in the pre‐gel mixture, gravity caused it to sag. Additionally, multiple branches were observed near the top of the glass substrate. As the PEGDA gel was pulled downward during photopolymerization, the laser beam's optical paths diverged, potentially leading to the formation of branches in the PEGDA fibers. The confocal length of the laser beam, within which diffraction effects of the focused beam are negligible, was also considered, as shown in Table 1. The confocal length of the Gaussian beam (86 µm) was smaller than the thickness of the pre‐gel solution (348 µm), determined by the parafilm spacer. This suggests that the wavefront curvature beyond the confocal region of the laser beam during photopolymerization may have contributed to the branching observed near the top glass substrate in Figure 3b. As expected, no helical PEGDA gel structure was observed when *l* = 0.

The PEGDA gels photopolymerized using optical vortex beams (*l* = +1, +4) exhibited different 3D structures compared to the case of *l* = 0. Specifically, the two ends of the PEGDA gels (attached to the top and bottom glasses) were located at similar positions on planes orthogonal to the laser irradiation direction, unlike in the case of *l* = 0 (Supporting Information, Figure S2). Moreover, the PEGDA gels were bent between the glass surfaces rather than perpendicularly (Video S2). These observations suggest that the OAM of the optical vortex beam “twisted” the PEGDA gels during photopolymerization. Two possible mechanisms for light‐induced helical fiber formation have been proposed. The first is the OAM driven optical rotation model in submicron‐scale particles as nucleation of polymerization.^[^ ^15^ ^]^ Second is the light‐induced self‐trapping/soliton effect model based on nonlinear refractive index change via photopolymerization,^[^ ^9^ ^]^ which manifests the trajectory of the Poynting vector of irradiated optical vortex. However, the origin of this phenomenon has not yet been definitely established. The direct observation of helical fiber formation by employing a high‐speed camera will be needed to identify the mechanism, however, it was prevented by the frosted side walls of the sample cell used in this experiment.

The current study does not aim to elucidate the mechanism of helical fiber formation, but to demonstrate helical biocompatible polymer fibers with optical vortex illumination. When using optical vortices with *l* of +2 and +3, the photopolymerized PEGDA gels exhibited a structure almost identical to that of the fiber with an opitcal vortex with *l* = +4 (Supporting Information, Figure S3).

The length of the helical fibers is another critical factor to be considered. The confocal length of the optical vortex beam was M^2^ times longer than that of the Gaussian beam, which proved advantages in this study. A longer confocal length of the optical vortex beam allowed the fabrication of longer gels with consistent diameters along their length. The confocal length of the optical vortex beam with *l* = +4 in this study was estimated to be 430 µm. This confocal length exceeds the cell thickness, thus resulting in the limitation of fabricated fiber length. Tissue engineering scaffords will require the extension of PEGDA fibers; for instance, photopolymerization with high‐order Bessel beams will allow the creation of larger helical tissues suitable for more expansive applications. According to the light‐induced self‐trapping/soliton effect model,^[^ ^9^, ^15^ ^]^ the fiber branching effects, in which a higher order optical vortex breaks up into single‐charged optical vortices to form a bundle of helical microfiber, occur in the process of transverse modulation instability of the incident optical vortex field. The incident laser power in this experiment was controlled to 0.8–0.9 µW. This value was < 1/1000^th^ of that (∼3 mW) in our previous work with NOA63, thus suppressing the fiber branching effects owing to modulation instability. Also, the refractive index change of PEGDA gel before and after photopolymerization was undetectably small (smaller than 0.01) in comparison with that (∼0.04) of NOA 63. Thus, the fiber branching effects was not observed significantly.

**Figure 4:**
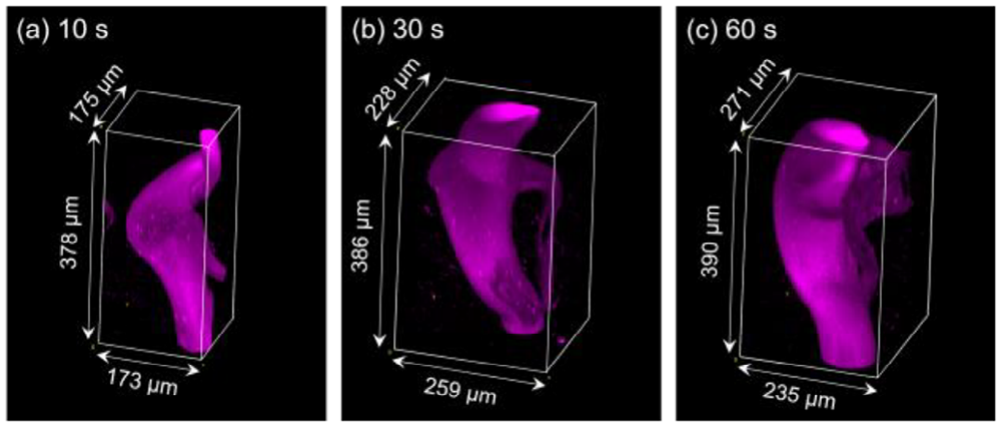
(a–c) Confocal fluorescence images of the PEGDA gels fabricated by *l* = +4 optical vortex beam irradiation for different durations. Each value on the top left indicates the photopolymerization duration. The laser power was 0.82 µW. The PEGDA gels were placed on the confocal microscope in the same manner as illustrated in Figure 3a. (a) is the same PEGDA gel in Figure 3c, which has been located here as well for comparison with (b) and (c).

**Figure 5:**
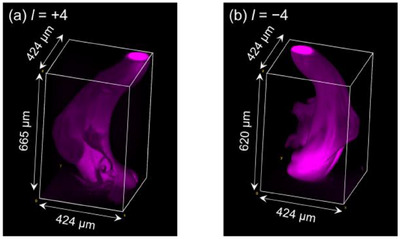
Confocal fluorescence images of the PEGDA gels fabricated by (a) *l* = +4 and (b) *l* = −4 optical vortex beams irradiation. Irradiation duration was 60 s. The laser power was 1.2 µW.

Photopolymerization was additionally conducted using an optical vortex beam with *l* = +4 for longer irradiation durations (30 and 60 s). As shown in Figure 4b,c (rotation of the PEGDA gels in Video S3) and Figure S4 the curved structure and positional overlap of the two ends were consistent with those of PEGDA gels photopolymerized with 10 s irradiation (Figure 4a). The cross‐sectional areas at different heights were calculated for each *l* = +4 PEGDA gel using z‐stack images (Figure S5a). Generally, the cross‐sectional area at each height increased as the irradiation duration increased. However, no consistent trend in cross‐sectional area changes with height was observed. The volumes of the PEGDA gels (Figure S5b) were proportional to the duration of laser irradiation. Longer irradiation durations generated more radical species, leading to increased formation of PEGDA clusters. These clusters crosslinked to form larger PEGDA gels, resulting in greater volumes for longer irradiation durations. Additionally, PEGDA gels were fabricated using *l* = +4 and −4 at increased laser power (1.2 µW, irradiation duration: 60 s) in a thicker pre‐gel solution, as depicted in Figure 5 and Video S4. Although determining whether the two gels possess opposite helical directions was challenging in this study, the results demonstrate that various structural properties of the photopolymerized PEGDA gels can be altered simply by controlling laser parameters. The use of optical vortex beams offers significant advantages, as they enable the creation of helical structures with precise control over laser parameters and beam positioning, providing the user with a versatile design for biomaterial applications.

## 3 Conclusion

We demonstrated the photopolymerization of biocompatible PEGDA gel fibers on cover glass using an optical vortex beam with a characteristic annular intensity profile. The PEGDA fibers exhibited helical structures only when fabricated using optical vortex beams with topological charges *l* = +1 or ± 4 (Figure 3c,d). The formation of these helical structures is attributed to the transfer of OAM to the photopolymerized PEGDA, as explained by the optical soliton model through self‐focusing effects or the generation of optical forces acting on the photopolymerized particles. In contrast, PEGDA fibers photopolymerized using a Gaussian beam (*l* = 0) exhibited cylindrical symmetry around the propagation axis without helicity (Figure 3a). The ability to easily control optical parameters, such as topological charge, laser power, and irradiation duration, makes this system highly versatile for engineering various helical PEGDA fibers. This capability enables the investigation of how helical structures influence cellular tissues. Furthermore, the versatility of this system allows the fabrication of helical fibers with various other polymeric materials, such as gelatin‐methacrylate^[^ ^16^ ^]^ (a cell‐adhesive natural polymer), or with different chemistries, including thiol‐ene reactions,^[^ ^17^ ^]^ as long as the polymerization reactions are photo‐mediated. This study highlights the potential of optical vortex beams for creating helical biomaterials and opens new avenues for applications in tissue engineering and regenerative medicine.

## Supporting Information

Supporting Information is available from the Wiley Online Library or from the author.

## Conflict of Interests

The authors declare no conflicts of interest.

## Supporting information



Supporting Information

Supporting Video 1

Supporting Video 2

Supporting Video 3

Supporting Video 4

## Data Availability

The data that support the findings of this study are available from the corresponding author upon reasonable request.
