# Supplementary material for: Fabrication of Biocompatible Helical Fibers Using an Optical Vortex Beam
Source: Chem Asian J. 2025 Jun 10;20(17):e00361. doi: 10.1002/asia.202500361 (PMC12447858; doi:10.1002/asia.202500361)
Supplement: Supplementary file 1 — Supporting Information [file ASIA-20-e00361-s004.docx]

Supporting Information

Fabrication of biocompatible helical fibers using an optical vortex beam

*Kenta Homma,^[a,b]^ Yoshihisa Matsumoto,^[c]^ Yasushi Tanimoto,^[c]^ Kyoko Masui,^[c]^*

*Chie Hosokawa,*^[c]^ Takashige Omatsu,*^[d,e]^ and Michiya Matsusaki*^[a]^*

1. Department of Applied Chemistry, Graduate School of Engineering, Osaka University, 2-1 Yamadaoka, Suita, Osaka 565-0871, Japan
2. Frontier Research Base for Global Young Researchers, Graduate School of Engineering, Osaka University, 2-1 Yamadaoka, Suita, Osaka 565-0871, Japan
3. Department of Chemistry, Graduate School of Science, Osaka Metropolitan University, 3-3-138 Sugimoto, Sumiyoshi-ku, Osaka 558-8585, Japan
4. Graduate School of Engineering, Chiba University, 1-33 Yayoi-cho, Inage-ku, Chiba 263-8522, Japan
5. Molecular Chirality Research Center, Chiba University, 1-33 Yayoi-cho, Inage-ku, Chiba 263-8522, Japan

**Materials**

Lithium phenyl-2,4,6-trimethylbenzoylphosphinate (LAP) and (3-trimethoxysilyl)propyl methacrylate were purchased from Tokyo Chemical Industry (Tokyo, Japan). Poly(ethylene glycol) diacrylate (PEGDA, number average molecular weight 6,000 g mol^-1^) and rhodamine B isothiocyanate were purchased from Sigma-Aldrich (MO, USA). Methanol was purchased from the Kishida Chemical Company (Osaka, Japan). 22 mm × 22 mm cover glasses and 24 mm×50 mm glass substrates (thickness: No.1, 0.13–0.17 mm) were purchased from Matsunami glass industry (Osaka, Japan).

**Optical Setup**

The photopolymerization of the PEGDA gels was conducted using the optical setup illustrated in **Figure 2(a)**. A continuous-wave laser beam with a wavelength of 405 nm (TEM-F-405-30 mW, Changchun New Industries Optoelectronics Technology, Jilin, China) was passed through a polarizing beam splitter to selectively extract p-polarized light. The laser beam was then expanded by two lenses and converted into an optical vortex beam (topological charge *l* = +1, ±4) passed through the spiral phase plate (VL-253-S-Y-A, HOLO/OR, Ness Ziona, Israel). The laser beam was focused using a × 5 objective lens (× 5, N.A. 0.1, MPlan; Olympus, Tokyo, Japan). The transmitted laser beam was collected using a × 20 objective lens (× 20, N.A. 0.3, LUCPlanFL N; Olympus, Tokyo, Japan) and captured with a CCD camera (acA1300-200uc; Basler, Ahrensburg, Germany). The power of the laser beam focused by the × 5 objective lens was measured using a laser power meter (StarLite, Ophir Optronics Solutions, Rehovot, Israel) and adjusted using an ND filter.

**Analysis method of a 405 nm laser beam**

The transmitted image of each laser beam at the focus was opened using ImageJ (National Institutes of Health (NIH), MD, USA), and the intensity profile along a line drawn through the center of the laser beam was analyzed. The fitting curves were constructed using the following Gaussian function:

$$\text{ }\text{I}\text{ }\left( \text{x} \right)\text{ = }\text{I}_{\text{0}}\text{ exp}\left( \text{-}\frac{\text{x}^{\text{2}}\ln\text{2}}{\text{r}^{\text{2}}} \right)$$

Here, *I* (*x*), *I*_0_, *x*, *r* represent the position-dependent laser intensity, peak laser intensity, position from the peak laser intensity, and half-width at half-peak laser intensity, respectively. The diameter of each laser beam was calculated using the fitting curve shown in Fig. 2(b). The theoretical diameter of each laser beam was calculated. For the Gaussian beam (*l* = 0), the following equation was used: theoretical diameter 2*W*_0_ = 1.22 × *λ* / N.A., where *W*_0_ is the beam radius at focus, *λ* is the wavelength of the laser beam (405 nm), and the N.A. is the numerical aperture of the × 5 objective lens used to focus the laser beam (N.A. 0.1). For the theoretical diameters of the optical vortex beams (*l* = +1 and +4), the scaling factor of $\sqrt{\text{l}\text{+1}}$ was multiplied by the theoretical diameter of the Gaussian beam.

The Rayleigh length *z*_R_ is the distance along the propagation direction of the laser beam from the focal point, where the beam diameter is increased by a factor of $\sqrt{\text{2}}$. The confocal length was twice Rayleigh’s length. Incorporating the *M* ^2^ parameter ( beam propagation ratio) of the laser source, the confocal length of each laser beam was calculated using the following equation:

$$\text{Confocal length}\text{ }\text{=}\text{ }\text{2 }\text{z}_{\text{R}}\text{ = }\frac{\text{2 π}\text{ }\text{W}_{\text{0}}^{\text{2}}}{\text{λ}\text{ }\text{M}^{\text{2}}}$$

where, *W*_0_ is the experimental laser beam radius at the focus, and *λ* is the wavelength of the laser beam (405 nm). An *M* ^2^ value of 1.1 was used, based on the specifications of the laser source in this study (*M* ^2^ < 1.1).

**Silanization of the cover glasses**

To stabilize the fabricated PEGDA gels on the cover glasses during photopolymerization, 22 mm × 22 mm cover glasses were immobilized with the silane coupling reagent (3-trimethoxysilyl)propyl methacrylate (**Figure S1(f)**). The cover glasses were first immersed in methanol for washing and then dried using a stream of nitrogen gas. The cover glasses were then treated with oxygen plasma (YHS-R; Sakigake-Semiconductor Company, Kyoto, Japan) for 5 min to activate the silanol groups. Immediately after the plasma treatment, the cover glasses were immersed in methanol (12.7 mL) containing 38.1 μL (3-trimethoxysilyl)propyl methacrylate and reacted for 1 h at room temperature. After the reaction, the silanized cover glasses were washed with methanol and dried under vacuum at 60 °C for 1 h in a vacuum oven (VOS-210C, Tokyo Rikakikai Company, Tokyo, Japan). The dried cover glasses were collected and stored at room temperature until further use.

**Photopolymerization of the PEGDA hydrogels by an optical vortex beam**

Prior to photopolymerization, a pregel solution chamber was created, as illustrated in **Figure 2(a)**. Specifically, a 22 mm × 22 mm silanized cover glass was mounted on a 24 mm × 50 mm glass substrate with three or four layers of Parafilm between the glasses such that a pre-gel mixture could be added to the space between the glasses. The chamber was heated in a drying oven (DG401, Yamato Scientific Company, Tokyo, Japan) at 70 °C to glue the glass and parafilm.

For the preparation of pre-gel mixture, 50 mg PEGDA (8.3 × 10^-6^ mol) and 7.4 mg LAP (2.5 × 10^-5^ mol) were mixed in 500 μL MilliQ containing 4.2 × 10^-2^ mg rhodamine B isothiocyanate (7.8 × 10^-8^ mol). The mixture was heated in an oven at 70 °C for 20 min to dissolve the reagents. Then, approximately 100 μL of the pre-gel mixture was filled in between the two glasses of the aforementioned chamber. As illustrated in **Figure 2(a)**, the chamber containing the pre-gel mixture was positioned in the optical setup so that the × 5 objective lens focused the laser beam on the interface between the 22 mm × 22 mm cover glass and the pre-gel mixture. Photopolymerization was conducted on the pre-gel mixture under specific conditions: topological charges (*l* = 0, +1, +2, +3 or ±4), laser power (0.8–0.9 µW or 1.2 µW), and laser irradiation duration (10, 30, or 60 s), as described in the main text.

**Fourier transform infrared spectroscopy measurement of the bulk PEGDA hydrogels**

Infrared spectra of PEGDA monomer and PEGDA gel were recorded using the Fourier transform infrared spectrometer (FREEXACT-II, Horiba, Kyoto, Japan) equipped with an attenuated total reflectance module (DuraSamplIR II, Smiths Detection, United Kingdom). PEGDA monomer was directly mounted on the spectrometer for the measurement. For the measurement of the PEGDA gel, 50 mg PEGDA (8.3 × 10^-6^ mol) and 7.4 mg LAP (2.5 × 10^-5^ mol) were first mixed in 500 µL MilliQ. A 50 µL pre-gel solution was photoirradiated with 405 nm light (1.0 × 10^3^ mW cm^-2^, equivalent to the laser intensity of *l* = +4 optical vortex), then immersed in MilliQ overnight to remove any unreacted monomer and photoinitiator from the fabricated PEGDA gel. The PEGDA gel was dried using a freeze-dryer (FDU-2200, EYELA, Tokyo, Japan). The dried PEGDA was mounted on the spectrometer to measure the IR spectra.

**Measurement of the rheological properties of the bulk PEGDA hydrogels**

For a viscoelastic material, its rheological properties can be measured by the stress response against an oscillatory shear strain *γ* = *γ*_0_ sin *ωt* (γ_0_: amplitude, *ω*: angular frequency, *t*: time). The stress response *σ* can be represented as *σ* = *σ*_0_ sin (*ωt* + *δ*) (*σ*_0_: amplitude, *δ*: phase angle, 0 < *δ* < π/2). This stress response can be further converted to:

$$\text{ }\text{σ}\text{ = }\left( \frac{\text{σ}_{\text{0}}}{\text{γ}_{\text{0}}}\cos\text{δ} \right)\text{γ}_{\text{0}}\sin\text{ωt}\text{ + }\left( \frac{\text{σ}_{\text{0}}}{\text{γ}_{\text{0}}}\sin\text{δ} \right)\text{γ}_{\text{0}}\sin\left( \text{ωt}\text{ + }\text{π}/\text{2} \right)$$

Here, the storage modulus *G*’ (represents the elastic property) and the loss modulus *G*’’ (represents the viscous property) are defined as:

$$\text{G}^{\text{'}}\text{ = }\frac{\text{σ}_{\text{0}}}{\text{γ}_{\text{0}}}\cos\text{δ}\text{, }\text{G}^{\text{''}}\text{= }\frac{\text{σ}_{\text{0}}}{\text{γ}_{\text{0}}}\sin\text{δ}$$

Rheological properties of the PEGDA before and after the photopolymerization were measured by a rotary shear rheometer (MCR302, Anton Paar, Austria) equipped with a glass peltier element (P-PTD200/GL, Anton Paar, Austria) and a 405 nm spot light source (LC-L1 V5, Hamamatsu Photonics, Shizuoka, Japan). 50 mg PEGDA (8.3 × 10^-6^ mol) and 7.4 mg LAP (2.5 × 10^-5^ mol) were first mixed in 500 µL MilliQ. A 50 µL pre-gel solution was filled into the 1 mm space between the rheometer stage and an 8 mm parallel plate. The storage modulus *G*’ and the loss modulus *G*’’ were constantly measured by oscillatory rheology at constant strain (1%) and frequency (1 rad s^-1^), during the 10 s photoirradiation to the PEGDA pre-gel solution at 1.0 × 10^3^ mW cm^-2^ (maximum intensity), which was equivalent to that of the *l* = +4 optical vortex laser beam. The moduli before and after the photoirradiation were extracted for comparison.

**Measurement of the refractive index**

The Abbe refractometer (DR-A1-Plus, ATAGO, Tokyo, Japan) was employed to measure the refractive index of the PEGDA pre-gel mixture and the bulk PEGDA hydrogel. For the measurement of the pre-gel mixture, 50 mg PEGDA (8.3 × 10^-6^ mol) and 7.4 mg LAP (2.5 × 10^-5^ mol) were mixed in 500 µL MilliQ. The mixture was heated in an oven at 70 °C for 20 min to dissolve the reagents. Then, the pre-gel mixture was cast on the refractometer and its refractive index was measured at 22 °C. For the measurement of the bulk PEGDA hydrogel, the pre-gel mixture was photopolymerized for 10 s using a 405 nm spot light source (LC-L1 V5, Hamamatsu Photonics, Shizuoka, Japan) at 1.0 × 10^3^ mW cm^-2^. The PEGDA hydrogel was placed on the refractometer and its refractive index was measured at 22 °C.

**Observation of the PEG hydrogels by confocal microscope**

After photopolymerization, the PEG hydrogels were washed with MilliQ water, and fresh MilliQ water was added to the chamber to prevent drying. The chamber containing the PEG hydrogels was placed on a confocal microscope (FV3000, Olympus, Tokyo, Japan). Z-stack images of the PEG hydrogels were captured using fluorescence observation (excitation wavelength: 561 nm, detection wavelength: 570–670 nm) of the rhodamine B encapsulated within the hydrogels. The step size for z-stack imaging was 2.00 µm for **Figures 3 and 4**, and 5.00 µm for Figure 5, using a × 30 objective lens (N.A. 1.05, UPLSAPO30XS, Olympus, Tokyo, Japan). The ImageJ software was used to reconstruct the three-dimensional structure of the PEG hydrogels. The surface area of each hydrogel at different heights was calculated from the z-stack images. The total volume of each hydrogel was determined by summing the products of the surface area and the z-stack step size.

**Absorbance measurements of PEGDA, LAP, and rhodamine B**

The absorbance spectra of PEGDA, LAP, and rhodamine B isothiocyanate were individually measured using an ultraviolet-visible absorption spectrometer (V-670, Japan Spectroscopic Corporation, Tokyo, Japan) equipped with a Peltier thermostated cell holder (ETCS-761, Japan Spectroscopic Corporation, Tokyo, Japan) and a circulation bath (MCB-100, Japan Spectroscopic Corporation, Tokyo, Japan). MilliQ containing 1.0 mg mL^-1^ PEGDA, 1.0 mg mL^-1^ LAP, or 1.4 × 10^-2^ mg mL^-1^ rhodamine B isothiocyanate was prepared. The solution was added to a quartz cell (optical path length: 1 cm), and its absorbance was measured in the range 300 nm–600 nm at 25 °C.

**Fluorescence measurement of rhodamine B**

MilliQ containing 1.4 × 10^-2^ mg mL^-1^ rhodamine B isothiocyanate was prepared and added to a quartz cell (optical path length: 1 cm). The fluorescence spectrum of rhodamine B from 300 nm–700 nm (excitation wavelength: 561 nm) was measured at 25 °C using a fluorospectrometer (FP-8500, Japan Spectroscopic Corporation, Tokyo, Japan) equipped with a Peltier thermostatted cell holder (ETC-815, Japan Spectroscopic Corporation, Tokyo, Japan) and a circulation bath (MCB-100, Japan Spectroscopic Corporation, Tokyo, Japan).

**Supporting Figures**


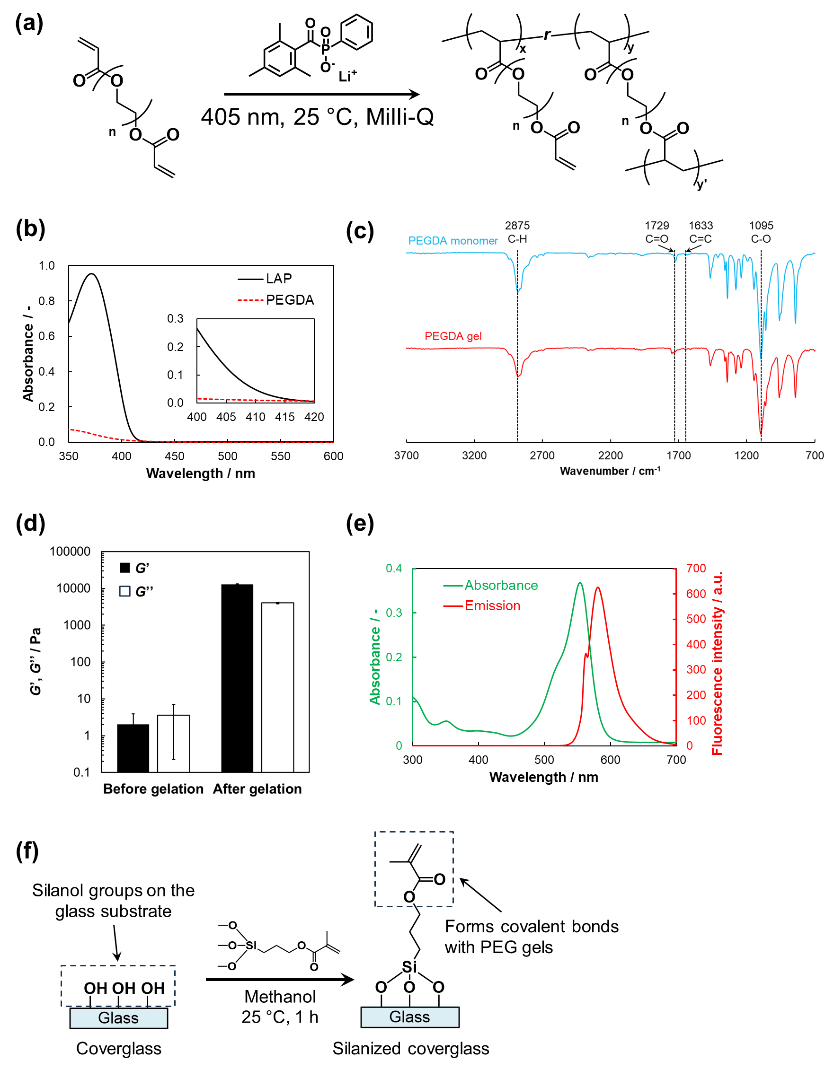


**Figure S1** (a) Reaction scheme for the photocrosslinking of poly(ethylene glycol) diacrylate (PEGDA). (b) The absorbance spectra of 1.0 mg mL^-1^ LAP (in a black solid line) and 1.0 mg mL^-1^ PEGDA (in a red dotted line) dissolved in MilliQ. LAP has an absorption at 405 nm, which allows the radical species to be formed by laser beam irradiation at this wavelength. (c) Fourier-transform infrared spectroscopy measurements conducted on PEGDA monomer and a bulk PEGDA hydrogel. The dotted line indicates the wavenumbers of specific bonds. (d) Rheological properties of PEGDA pre-gel solution (before gelation) and bulk PEGDA gel (after gelation), measured with a rotary rheometer. The laser intensity used for the photopolymerization was 1.0 × 10^3^ mW cm^-2^. The error bars represent the standard deviation. (e) The absorbance (in green line) and fluorescence (in red line) spectra of 0.14 mg mL^-1^ rhodamine B isothiocyanate (excitation wavelength: 561 nm) in MilliQ. The minor peak at 560 nm in the emission spectra is from the leaked excitation light. (f) Modification of the silane coupling reagent (3-trimethoxysilyl)propyl methacrylate) on the surface of the cover glass. The methacrylate groups at the terminal create covalent linkages with the PEG gels, which stabilize the fabricated gels on the glass substrate.


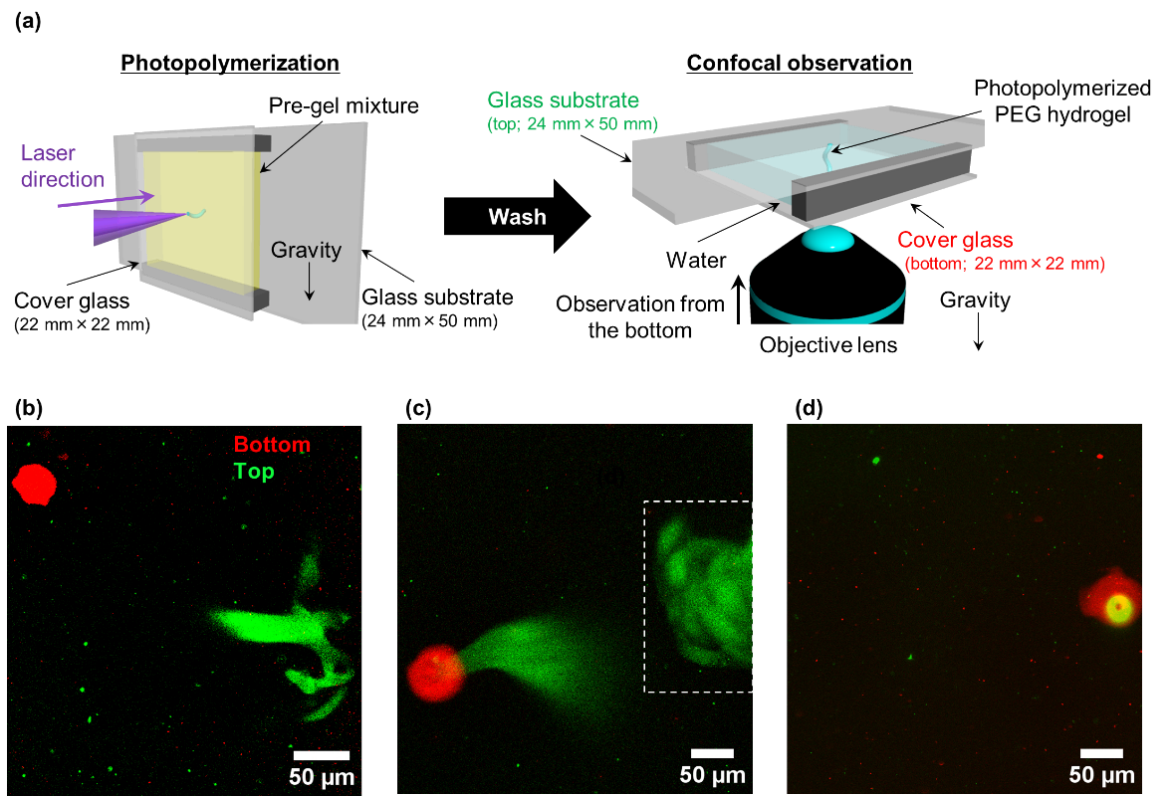


**Figure S2** (a) Confocal images of the photopolymerized PEG hydrogels from Figure 3 observed at bottom and top glass substrates. (b-d) The confocal images taken at the bottom cover glass (in red) and the top glass substrate (in green) were merged to show the overlap of the PEG hydrogel positions at the two ends. The images are from the PEG gels photopolymerized with (b) Gaussian beam (*l* = 0), and optical vortex beam with (c) *l* = +1 (the green gel in the dotted rectangle is another gel) and (d) *l* = +4. The laser irradiation duration was 10 s.


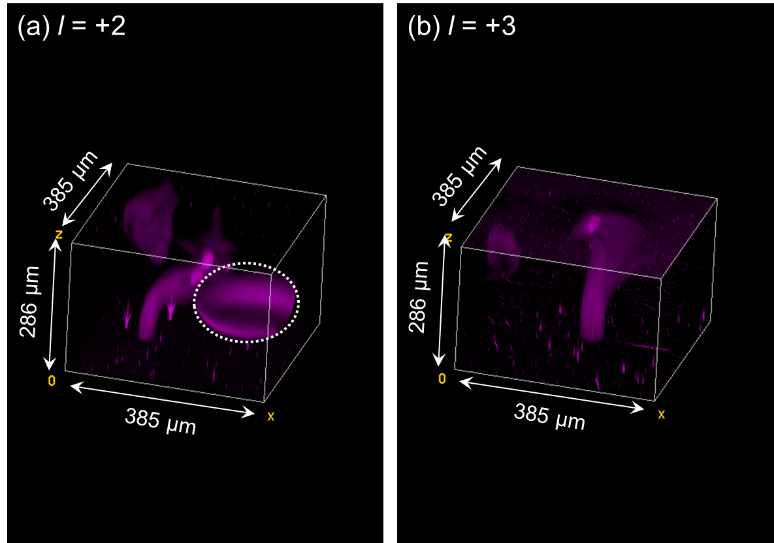


**Figure S3** Confocal fluorescence images of the PEGDA gels fabricated by (a) *l* = +2 and (b) *l* = +3 optical vortex beam irradiation for 10 s. The fluorescence from inside the dotted circle in (a) is a bubble.


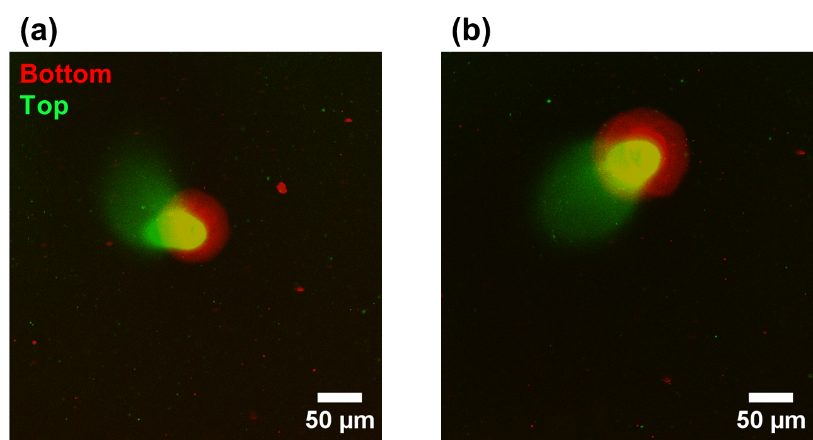


**Figure S4** Similar to Figure S2, confocal images of the photopolymerized PEG gels from Figure 4 were observed at bottom and top glasses. (a, b) The confocal images taken at the bottom cover glass (in red) and the top glass substrate (in green) were merged to show the overlap of the PEG gel position at the two ends. The photos are from the PEG gels photopolymerized with *l* = +4 optical vortex beam, for different laser irradiation duration: (a) 30 s, (b) 60 s.


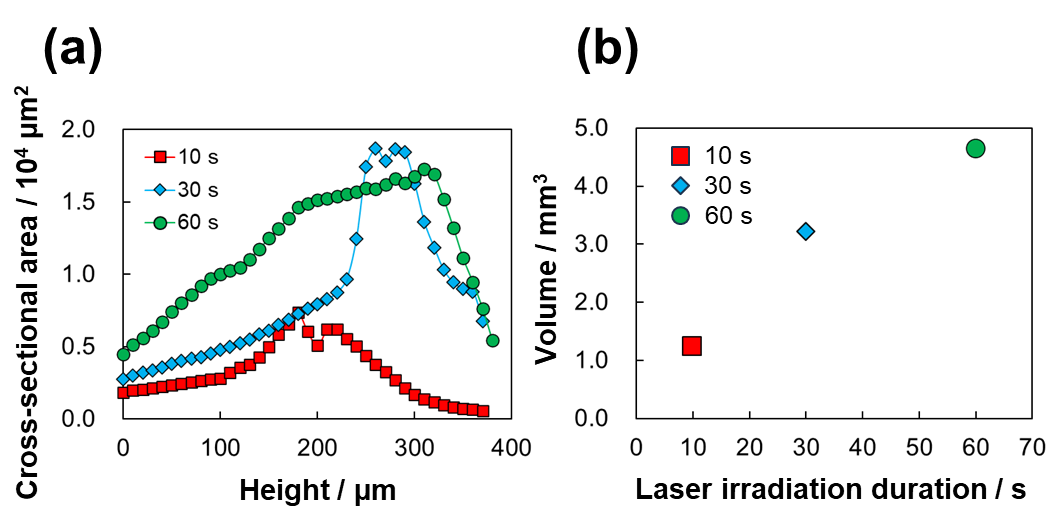


**Figure S5** (a) Cross-sectional area of the PEG gels (in Figure 4, photopolymerized using *l* = +4 optical vortex beam) at different heights. The values in the legend represent the photopolymerization duration of each PEG gel. (b) The volume of each PEG gel fabricated in Figure 4 by *l* = +4 optical vortex beam.

**Video S1** Time-lapse observation of the transmitted 405 nm optical vortex beams with different topological charges (*l* = +1, +4) during photopolymerization of PEG gels (laser irradiation duration: 10 s). The transmitted light was immediately distorted after the onset of photoirradiation, indicating that the formation of a photocrosslinked structure was induced within seconds of irradiation. The videos were displayed in real time.

**Video S2** Three-dimensional structures of representative PEG gels photopolymerized using laser beams with different topological charges (*l* = 0, +1, and +4) under 10 s of laser irradiation. The PEG gel images obtained by confocal microscopy were rotated. The PEG gels were photopolymerized by laser irradiation from the bottom in all the figures. The height in each figure represents the thickness of the pregel solution (equal to the distance between the bottom and top cover glasses). A scale bar on the bottom right was applied to all three gels. The label on each PEG gel indicates the topological charge of the laser beam used to photopolymerize the gel.

**Video S3** Three-dimensional structures of representative PEG gels photopolymerized using *l* = +4 optical vortex beams under different irradiation durations (10, 30, and 60 s). The PEG gel images obtained by confocal microscopy were rotated. The PEG gels were photopolymerized by laser irradiation from the bottom in all the figures. The height of each figure corresponds to the thickness of the pregel solution. A scale bar on the bottom right was applied to all three gels. The label on each PEG gel indicated the duration of laser irradiation for photopolymerization.

**Video S4** Three-dimensional structures of the representative PEG gels photopolymerized using *l* = ±4 optical vortex beams under the 60 s laser irradiation. The PEG gel images obtained by confocal microscopy were rotated. The PEG gels were photopolymerized by laser irradiation from the bottom in all the figures. The height of each figure corresponds to the thickness of the pregel solution. A scale bar on the bottom right was applied to both gels. The label on each PEG gel indicates the topological charge of the laser beam used to photopolymerize the gel.
